# Impact of Exercises and Chair Massage on Musculoskeletal Pain of Young Musicians

**DOI:** 10.3390/ijerph17145128

**Published:** 2020-07-16

**Authors:** Anna Cygańska, Aleksandra Truszczyńska-Baszak, Paweł Tomaszewski

**Affiliations:** 1 Faculty of Rehabilitation, Józef Piłsudski University of Physical Education in Warsaw, Poland, Marymoncka 34, 00-968 Warsaw, Poland; aleksandra.rapala@wp.pl; 2 Faculty of Physical Education, Józef Piłsudski University of Physical Education in Warsaw, Poland, Marymoncka 34, 00-968 Warsaw, Poland; pawel.tomaszewski@awf.edu.pl

**Keywords:** musculoskeletal pain, playing related musculoskeletal disorders, musician, physiotherapy

## Abstract

Due to the occurrence among musicians of musculoskeletal problems associated with playing a musical instrument, it is necessary to use prophylaxis. The aim of the study was to compare the effectiveness of two physioprophylaxis methods: chair massage and an original set of exercises. The study lasted four weeks and consisted of eight 15-min meetings (chair massage/exercises). The study was conducted on 44 music students assigned to three groups (chair massage/exercise program/control group). The algometric measurements and questionnaire were conducted. Health problems associated with playing an instrument was reported by 86.4% of the participants. The largest changes in pain threshold concerned the trigger points of the muscles with the highest pain sensitivity, i.e., upper part of trapezius ones, and reached 25–34% in relation to the initial values. For the trigger points of the levator scapulae and lower part of trapezius, the increase in the pain threshold was between 20 and 28%. Raising the pain threshold was observed both after each session and meeting by meeting, and these differences were most visible in the massage group. This effect was particularly visible from the fourth treatment. Chair massage and exercise should be used regularly, and significant results can be obtained after two weeks.

## 1. Introduction

Artists playing musical instruments, due to loads related to the strain of playing the instrument, suffer from various forms of playing related musculoskeletal disorders (PRMD) [1,2,3,4]. Most problems concern the musculoskeletal system; it is reported by up to 89% of musicians [5,6]. Studies show that health problems related with playing musical instruments more often concern women [1,5,7]. In addition, research shows that health problems also affect children learning to play instruments [8,9] and students [10,11,12]. Pain among artists is often recognized as chronic and occur as a result of cumulative, long-lasting overload [11] but it also can be an acute pain that is especially related to a sudden increase in instrument practice [13]. Analysis of these aspects shows that for young people in music schools and universities, the specific prevention programs that would guide musicians how to avoid pain and injury could be of great benefit for their condition and performance.

There are many forms of physioprophylaxis of musculoskeletal pain (relaxation exercises, body awareness exercises, stretching, stabilizing exercises, and massage). Specialists and scientists emphasize the need to develop prevention programs for this occupational group and they recommend physical exercises as the most efficient intervention [14,15,16,17,18]. Exercises should not be complicated, so that every musician is able to follow them properly after instruction and should include muscles and areas exposed to overload and pain. In selecting the appropriate form of prophylaxis in this occupational group, the challenge is to take into account the specificity of musicians’ work [19].

The music profession hinders regular use of physiotherapy sessions, which is why, according to the authors, a good form of prophylaxis is chair massage, because it can be performed at the place of work/stay of musicians. It is a treatment lasting from 15 to 20 min, in a special chair. During the treatment, the physiotherapist performs massage techniques, acupressure and stretching, focusing mainly on the area around the neck, head, upper limbs and back [20,21]. Chair massage as a form of prophylaxis has also been used among other professional groups exposed to musculoskeletal overload [21,22].

The aim of the study was to compare the effectiveness of two physioprophylaxis methods: chair massage and exercises in a group of student-musicians.

## 2. Methods

### 2.1. Study Population

After obtaining the consent of the Senate Ethics Committee for Scientific Research (SKE 01-61/2017), 44 students of The University of Music, comprised of 30 women and 14 men, were included in the study. Each participant was informed about the purpose and procedure of the research, as well as the possibility of resigning from participation at any time during the study. All participants gave their informed consent to participate in the project. The participants were constituted a convenience sample and were assigned to three groups: group I-massage on a chair-16 people, group II-exercises-14 people, group III (control group)-14 people. The groups did not differ in terms of weight, height, daily time of playing the instrument, duration of play in years, or reported pain, the results are presented in Table 1.

Participants reporting the occurrence of current disorders estimated their intensity at 2.5 ± 2.1 (on a VAS scale of 0–10). The participants played different instruments: 18 people played the violin/viola, 6 people played the cello, 4 people played bassoon, 3 played the accordion and trombone respectively, 2 played the French horn, oboe, flute, and harp, respectively, and 1 person played the double bass and percussion, respectively.

The qualification criterion for the study was: playing a musical instrument for a minimum of 5 years, while the disqualification criteria were: neurological and oncological diseases, medical contraindication to undertake physical exercise, contraindications to perform massage, and lack of consent for participation in the study.

### 2.2. Physioprophylaxis Methods

The original set of exercises was developed based on the knowledge and clinical experience of the authors of the study and based on EBP (evidence based practice). The program consisted of 14 strengthening and stretching exercises involving the muscles of the trunk and limbs whose main goal was to mobilize the joints and strengthen the weakened muscles and relax overloaded muscle groups. Exercises were performed in the following position: sitting in a chair (anteversion and retroversion of the pelvis-20 repetitions (reps.), alternating trunk twists right and left-10 reps., retraction of the cervical spine and head-10 reps., lifting and lowering the shoulder girdle-20 reps., backward shoulder girdle rotation-10 reps., alternating pro- and supination of the forearms-10 reps., rotation in the wrist joints-10 reps.), supine position (lifting the trunk and upper limbs with simultaneous retraction of shoulder girdle-30 reps.), prone position (thigh extension in hip joint-20 reps. per side), the bridge exercise-30 reps., the dead bug exercise-30 reps. per side, hamstring stretching-3 reps. (30 s per side), and in a standing position (stretching of the thoracic girders, resting one upper limb on the door frame-3 reps. (30 s per side)).

**Figure 1:**
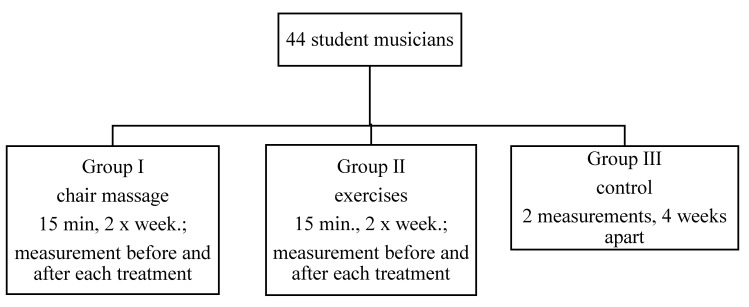
Study design.

Chair massage was performed on the basis of the Work-Site Massage School. Only traditional methods of Swedish massage were used, consisting of stroking (smooth, continuous movements to relax and calm the sympathetic nervous system), kneading (consisting of wringing, squeezing muscles, compressing pressure points for the relaxation of soft tissues, i.e., muscles, tendons, and fascia), stretching combined with swaying and passive movements of the upper limbs (to mobilize the joints of the upper limbs), and final stroking combined with slapping and shaking percussion, vibration. All massages, exercises, and measurements were conducted and supervised by the same specialist (physiotherapist with 5 years of experience), and took place in the morning, in a quiet and comfortable room; at the request of the participants, no music was used during the treatments. The study program is presented in Figure 1.

### 2.3. Measurement Methods

The subjective evaluation was based on the Polish-language version of the Musculoskeletal Pain Intensity and Interference Questionnaire for Professional Musicians (MPIIQM-P) questionnaire (unpublished data-publication in progress); (questions about work in the orchestra were omitted-4, 6, and 8) and the author’s original subject log ended with a questionnaire for satisfaction with the proposed treatments. The MPIIQM-P questionnaire consists of 22 questions, divided into three parts: demographic data and information referring to playing the instrument, questions about the intensity of pain/problems, and questions about pain/problems interference.

Algometric measurements were made as an objective assessment of the treatments carried out before and after each therapeutic session. The algometric measurement concerned the assessment of muscle pressure sensitivity-pain threshold in kg/cm^2^ made using the Pain Test FPX Algometer (Wagner). Algometric measurement is an effective computational tool for myofascial pain [23,24,25]. The measurements were made at 6 muscle trigger points on the right and left side of the body: trapezius (descending/upper part), levator scapulae, and trapezius (ascending/lower part). After palpating the trigger points of the mentioned muscles, according to Nie et al. 2005 methodology [26], they were marked with dots using a special marker. The measurement was always performed in the prone position (the face was placed in the table opening with upper limbs along the torso), applying the device at a 90-degree angle, always starting from the points on the right, as illustrated in Supplementary Figure 2. The patient was asked to say “stop” at the moment of feeling pain at the point under examination. The result was read by the investigator after the measurement. The temperature, noise, and lighting in the room were always constant.

### 2.4. Data Analysis

In the description of the results, basic statistical methods were used: arithmetic means, standard deviations (SD), standard errors (SE), and percentages. The normality assumption was verified using the Shapiro–Wilk test, the assumption of homogeneity of variances in the groups was tested by the Levene test. Both assumptions proved not to be violated, data were thus subjected to parametric procedures. The significance of changes in mean values of pressure sensitivity in the studied groups was assessed using the 3 × 2 (Group × Study) mixed-design (split-plot) analysis of variance (ANOVA), separately for the right and the left side of the body. Additionally, in the massage and exercise group, the assessment of the direct effect of the treatments applied during 8 meetings was performed using the 2 × 8 × 2 mixed-design ANOVA (Group × Study × Before/After). The assumption of sphericity was verified using the Mauchley test, in the case of significant violations, the Greenhouse–Geisser correction was applied. For significant ANOVA effects, detailed comparisons between pairs of means were made post hoc using the Tukey test. As a measure of the size of the effect, partial eta-square (η^2^) was used. The correlation between quantitative features was assessed using Pearson’s correlation. All analyses were performed using the STATISTICA 13 program (TIBCO Software Inc., Palo Alto, CA, USA). In the assessment of the significance of the effects, the p level was <0.05. Trial registration was on: ClinicalTrials.gov Identifier: NCT04366843, 28.04.2020 r.

## 3. Results

### 3.1. Questionnaire Results

The occurrence of a lifetime prevalence of PRMD was reported by *n* = 38 (86.4%) of the participants, and *n* = 29 (65.9%) of respondents reported the presence of current PRMD. The participants currently declaring the presence of PRMD did not differ in terms of BMI (*p* = 0.41), or seniority (*p* = 0.14), and intensity of playing the instrument (*p* = 0.08).

Of all participants reporting PRMD, 81.8% were women. Women declared (*p* = 0.014) a longer period of playing the instrument (13 ± 2.5 vs. 10.6 ± 3.4 years), with no significant differences between women and men in terms of age (*p* = 0.78). In contrast, men reported statistically significant (*p* = 0.047) more time per week devoted to playing the instrument at 29.6 ± 10.2 h vs. women 23.3 ± 9.0 h. In the women’s group, the most common location of pain was the cervical spine (36%), and in the men’s group problems were reported within the shoulder girdle and left arm (43%) and the lumbar spine (43%). There were no differences between men and women due to the pain intensity factor which was 12.93 ± 5.79 (*p* = 0.51) or the pain influence factor was 18.5 ± 11.8 (*p* = 0.47). None of the participants had PRMD in the head area and lower limbs.

Participants from groups I and II (*n* = 15) after the completed program assessed its usefulness at 8.8 ± 1.42 (on a scale of 0–10, where 0 was not useful and 10 was very useful, I will use it), whereas the impact of the program on reported PRMD was estimated at 6.7 ± 1.11 (*n* = 7). In none of the examined PRMD worsened, and currently complaints are reported by only four participants (two from the exercise group and two from the massage group). Due to the fact that few of the participants completed the follow up questionnaire in a reliable way, too little data was obtained to make statistical calculations.

### 3.2. Algometry Measurements Results

For none of the trigger points tested, the main effect of the group was found to be significant; thus, there were no significant differences between the groups of massage, exercise, and control group (η^2^ from 0.010 to 0.114). After the program, a reduction in the pressure sensitivity of the tested trigger points was observed in the groups subjected to treatment, the largest differences being in the massage group. Significant interaction was found for the trigger points of the right levator scapulae (F_2,39_ = 3.66, *p* = 0.035, and η^2^ = 0.158) and left (F_2,39_ = 6.70, *p* = 0.003, and η^2^ = 0.264) and the right lower part of trapezius muscle (F_2,39_ = 7.10, *p* = 0.002, and η^2^ = 0.267). It was also observed that in the massage and exercise groups the pressure sensitivity of the muscles decreased, and in the massage group this effect was greater (Table 2). At the same time, in the control group, an increase in the pressure sensitivity of these trigger points was observed. For the point located on the left upper part of trapezius, after the study and independent of group, a significant decrease in pressure sensitivity (F_1,39_ = 5.46, *p* = 0.025, and η^2^ = 0.1123) was observed, while for the right upper part of trapezius and left lower part of trapezius muscle trigger points, no significant differences were observed.

**Figure 3:**
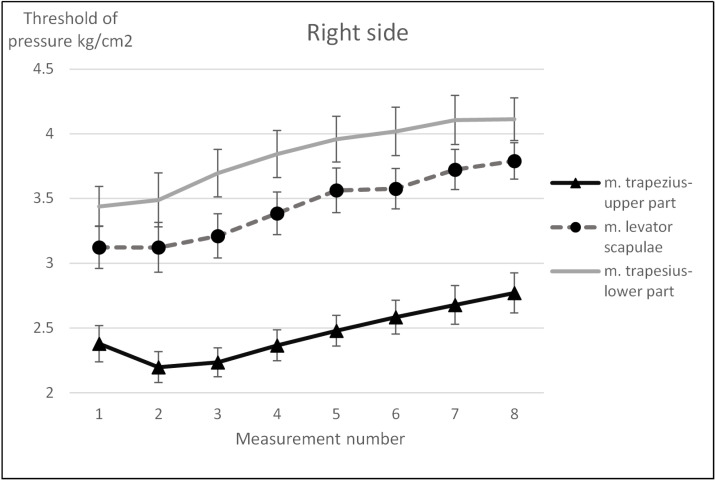
Changes in pain sensitivity of the examined muscle trigger points on the right side (Mean, SE).

**Figure 4:**
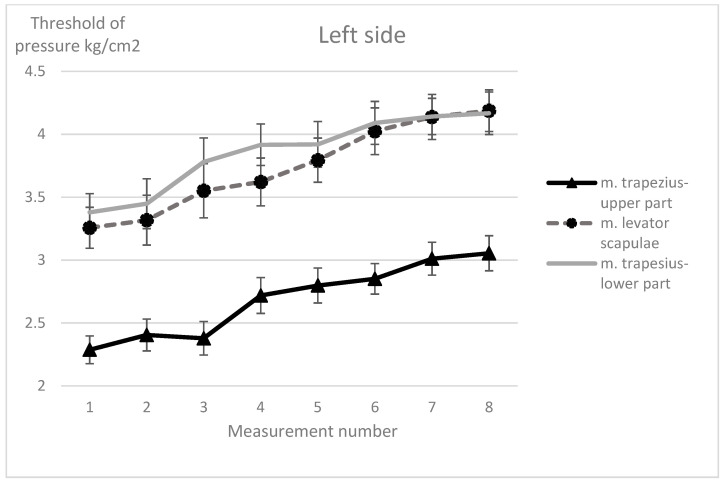
Changes in pain sensitivity of the examined muscle trigger points on the left side (Mean, SE).

For none of the trigger points examined, both for the right and left side, was there any significant Group × Study × Before/After (η^2^ from 0.010 to 0.072) interaction or Group × Study (η^2^ from 0.023 to 0.046) interaction. There were no significant differences between the groups (η^2^ from 0.001 to 0.136). Nevertheless, the pain threshold of all examined trigger points of the muscles increased (pain sensation decreased) immediately after each treatment (η^2^ from 0.478 to 0.736), as well as in subsequent treatments (η^2^ from 0.163 to 0.263). The largest changes concerned the trigger points of the muscles with the highest pain sensitivity, i.e., upper parts of trapezius muscles and reached 25–34% in relation to the initial values, for the trigger points of the levator scapulae and lower parts of trapezius muscles, the pain threshold increased by around 20–28% (Figure 3 and Figure 4).

In addition, for the right side upper part of trapezius and lower part of trapezius trigger points (respectively, F_1.25_ = 11.9, *p* < 0.001, and η^2^ = 0.323, and F_1.25_ = 4.49, *p* = 0.044, and η^2^ = 0.152) and on the left side (respectively, F_1,25_ = 4.86, *p* = 0.037, and η^2^ = 0.163, and F _1.25_ = 25.3, *p* < 0.001, and η^2^ = 0.503) in the massage group, a greater increase in the pain threshold was observed immediately after the treatment (reduced pain sensation) as compared to the exercise group (significant interaction Group × Before/After). On the other hand, for the following muscle trigger points: right levator scapulae (F_7,168_ = 2.36, *p* = 0.025, and η^2^ = 0.090), left upper part of trapezius (F_7,175_ = 2.48, *p* = 0.019, and η^2^ = 0.090), left lower part of trapezius (F_7,175_ = 2.22, *p* = 0.035, and η^2^ = 0.082), a significant Study × Before/After interaction was observed demonstrated by an increase in the pain threshold both immediately after the treatment and meeting to meeting. This dependence is particularly visible from the second treatment (third and fourth measurement), where the differences in the threshold of pain before and after the treatment were becoming more pronounced.

## 4. Discussion

Many artists complain about pain that appears during their education and work [7,27,28,29]. Scientific research confirms the need to implement the prophylaxis of musculoskeletal diseases among musicians [15,17,18,30,31]. There are various programs and trainings aimed at preventing musicians’ PRMD [32,33,34,35]; however, according to the authors of the study, creating a prevention program for young musicians should first of all take into account the nature of strains, the specificity of work and the location of complaints. The aim of the research was to compare the effectiveness of two methods of prophylaxis that meet the above criteria. Both proposed programs positively influenced the reported complaints. There was a decrease in the pressure sensitivity of the majority of the muscle trigger points examined; thus, confirming the validity of selecting these forms of prevention. The method recommended by the authors would be chair massage due to significantly greater improvement than using the exercises alone. Both forms of prophylaxis were very popular among the participants. At the same time, in the control group, an increase in the pressure sensitivity of these trigger points was observed. Probably the cause was that the study was performed during the second half of the term before the exams, when the intensity of playing was increasing.

An alarmingly high percentage of musicians declaring PRMD was reported, both over their entire life and in the last seven days, similar to research by Kochem and Silva [12] and Roos and Roy [36]. Women were shown to be more prone to PRMD, especially when it comes to the cervical spine area, which was also indicated by other researchers [12,36]. It was also observed that women declared a statistically significantly shorter time of playing the instrument per week as compared to men. This may be due to the fact that women limited the time of playing the instrument due to current complaints experienced.

Musculoskeletal disorders also include Craniomandibular Disorders or Temporomandibular Disorders (TMDs) [6]. Many authors investigating this issue often present discrepant results, and based on current evidence-based medicine standards systematic review conducted by van Selms et al. [37], clearly states that there is no scientific and unambiguous confirmation that playing a musical instrument poses a risk of developing TMD as an occupational disease for musicians. In their own study, musicians reported the presence of strongest pain in various parts of the body; however, none of the participants chose the area of the face or head as the area where pain is the strongest. This may indicate that the musicians’ awareness of the PRMD in this area is small, and that these PRMD are not so aggravating compared to others.

The results of algometric measurements indicate a positive effect of exercises on the level of pain threshold of the participants, but first and foremost, the chair massage should be used. The respondents from groups I and II positively assessed the applied treatments, evaluating the suitability of the program at around nine on a 10-point scale of assessment. Similar results were obtained by other researchers using this form of prophylaxis of musculoskeletal problems among other professional groups [21,38,39]. Special attention was paid to such benefits as reduction of stress, improvement of sleep comfort, reduction of pain, headaches, as well as reduction of tension and stress, the participants reported relaxation and inflow of energy, and increased mobility of the cervical spine [22,40].

As an enhancement to the proposed treatments, the authors believe that educational activities should also be incorporated. Because during the study of both massage and exercise students had many questions about the most common injuries, the reasons for their formation and how to cope independently with various types of PRMD, which was also pointed out in research by Arnson et al. [19].

The value of the research conducted is to propose a PRMD prophylaxis program taking into account the specificity of the work/education of musicians. For the first time, a chair massage was introduced as a form of prophylaxis. An important advantage of the study is the personal supervision of the participants during the whole program by a physiotherapist experienced in working with musicians; thus, ensuring the correctness of the program and confirming their execution, the verification of which is often difficult when participants after short instruction are supposed to do the exercises on their own.

### 4.1. Study Limitation

As a limitation of the study, it should be pointed out that due to the insufficient number of participants it was not possible to carry out the analysis for individual types of instruments. For organizational reasons, randomization was not possible. According to the authors, prophylaxis should have a general form, however it would be advantageous to think about compensatory exercises for individual types of instruments.

### 4.2. Recommendations

To obtain the best results in pain reduction in musicians the prevention interventions need to be applied regularly and for a long-lasting time (at least two weeks). Musicians can be subjected to chair massage or do the exercises but simultaneously they need to be supervised by a professional physiotherapist who would check and correct the execution of the exercises.

## 5. Conclusions

The results of the study indicate the validity of introducing chair massage as one of the forms of preventing musculoskeletal problems among musicians in institutions educating and employing artists. Both chair massage and exercise should be used regularly, and significant results can be obtained after approximately two weeks of use.

## Figures and Tables

**Figure 2:**
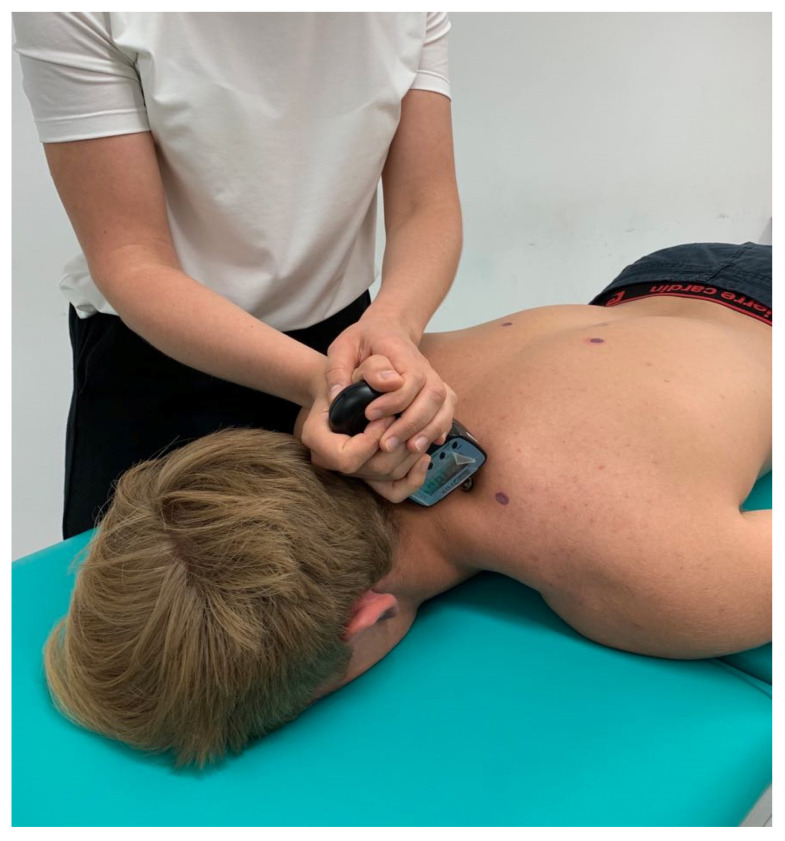
Algometric measurement of levator scapulae muscle trigger point.

**Table 1:** Participants characteristics (Mean, SD).

Parameter	Massage	Exercises	Control	*p*-Value
	(*n* = 16)	(*n* = 14)	(*n* = 14)	
Age (years)	20.6 ± 1.2	21.07 ± 2.0	20.1 ± 1.1	0.26
Body mass (kg)	58.7 ± 8.4	63.6 ± 12.9	61.48 ± 11.0	0.57
Body height (cm)	168 ± 6.9	170.4 ± 8.2	169.8 ± 6.8	0.78
Weekly playing time (hours)	24.8 ± 10.8	21.4 ± 10.4	24.1 ± 10.3	0.74
Duration of playing (years)	11.9 ± 3.4	11.9 ± 2.6	11.8 ± 2.6	0.63
Participants reporting current PRMD (n)	10	9	10	0.87

**Table 2:** Values of pressure sensitivity of selected muscle trigger points on the right and left sides measured in the study groups before and after the program (Mean, SD).

Variable	Massage (*n* = 16)		Exercises (*n* = 14)		Control (*n* = 14)	
	Before	After	Before	After	Before	After
Right Side						
m. trapezius (upper part)	2.3 ± 0.8	2.8 ± 0.9	2.3 ± 0.9	2.4 ± 0.8	2.8 ± 0.9	2.5 ± 1.0
m. levator scapulae ^**C**^	3.1 ± 0.9	3.8 ± 0.9	3.0 ± 1.0	3.4 ± 0.9	3.5 ± 0.9	3.1 ± 0.9
m. trapezius (lower part) ^**C**^	3.3 ± 0.8	4.0 ± 1.4	3.6 ± 0.9	3.9 ± 1.0	4.2 ± 1.2	3.5 ± 1.2
Left Side						
m. trapezius (upper part) ^**B**^	2.4 ± 0.7	3.0 ± 0.8	2.1 ± 0.4	2.6 ± 0.9	2.5 ± 0.7	2.5 ± 0.9
m. levator scapulae ^**B,C**^	3.3 ± 1.1	4.3 ± 0.9 *	3.0± 0.8	3.7± 0.8	3.5 ± 0.9	3.0 ± 1.0
m. trapezius (lower part)	3.4 ± 1.0	4.0 ± 0.7	3.3 ± 0.8	3.7 ± 1.0	4.1 ± 1.1	3.6 ± 1.1

B—Significant (*p* < 0.05) main effect Study; C—significant (*p* < 0.05) interaction effect Group x Study; * Significantly (*p* < 0.05) different from corresponding value observed prior to Study.
